# EHMTI-0017. Investigation of behavior of rats by nitroglicerine induced headache

**DOI:** 10.1186/1129-2377-15-S1-F4

**Published:** 2014-09-18

**Authors:** D Akcali, Y Sara, A Babacan, H Bolay

**Affiliations:** 1 Anesthesiology Algology Department Neuropsychiatry Center, Gazi University School of Medicine, Ankara, Turkey; 2 Pharmacology Department, Hacetepe University School of Medicine, Ankara, Turkey; 3 Anesthesiology Algology Department, Gazi University School of Medicine, Ankara, Turkey; 4 Neurology Algology Department Neuropsychiatry Center, Gazi University School of Medicine, Ankara, Turkey

## Introduction

Chronic migraine is a debilitating disease particularly in women and underlying pathophysiology remains unclear. Clinically relevant migraine models are missing.

## Aims

The aim of this study is to mimic chronic migraine model in rats and to test migraine drugs.

## Methods

Study was approved the Institutional Animal Care and Use Committee and care and handling of animals were in accord with National Institute of Health guidelines. Nasociliary nerve (NCN), which is the rat correlate of opthalmic branch of trigeminal nerve is ligated to mimic chronic headache. Glyceroltrinitrate (GTN) was administered to induce acute migraine attack. Sumatriptane and CGRP receptor antagonist were administered. Pain, anxiety related behavior were recorded. Mechanical allodynia, thermal hyperalgesia was tested by VF/ EVF and acetone; anxiety by elevated plus maze (EPM). Activated areas were investigated by c-fos immunoreactivity and plasma extravasation was studied by horse radish peroxidase.

## Results

NCN ligation, GTN administration model demonstrated mechanical allodynia, thermal hyperalgesia. Anxiety accompanying pain was confirmed by EPM. Extravasation in dura mater was shown by Horse radish peroxidase. Significantly c-fos immunoreactivity was increased in ipsilateral brainstem TNC compared to contralateral and also in cortical structures constituting pain matrix. CGRP antagonists decreased pain related behavior and c-fos positive cells.

## Conclusions

Mechanical allodynia, thermal hyperalgesia, c-fos staining confirming central and peripheral sensitization is exhibited in NCN ligated rats and pain related anxiety is confirmed. CGRP receptor antagonists are effective for chronic headache treatment. This chronic migraine model is relevant to human migraine and eligible for further drug investigations.

No conflict of interest.

